# Supplementary material for: PIP4K2C inhibition reverses autophagic flux impairment induced by SARS-CoV-2
Source: bioRxiv. 2024 Apr 17:2024.04.15.589676. Preprint. [Version 1] doi: 10.1101/2024.04.15.589676 (PMC11042293; doi:10.1101/2024.04.15.589676)

Supplementary Fig. 1

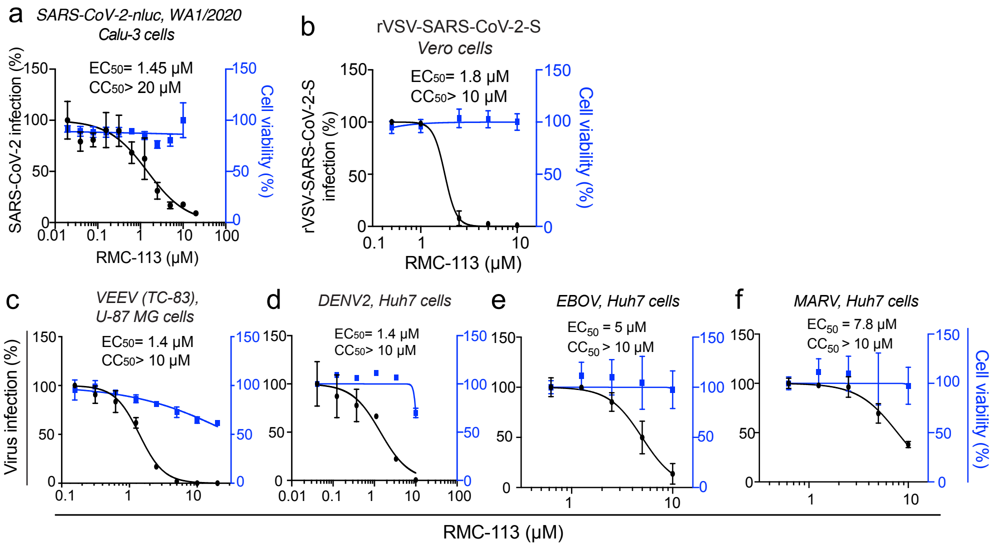

Supplementary Fig. 2

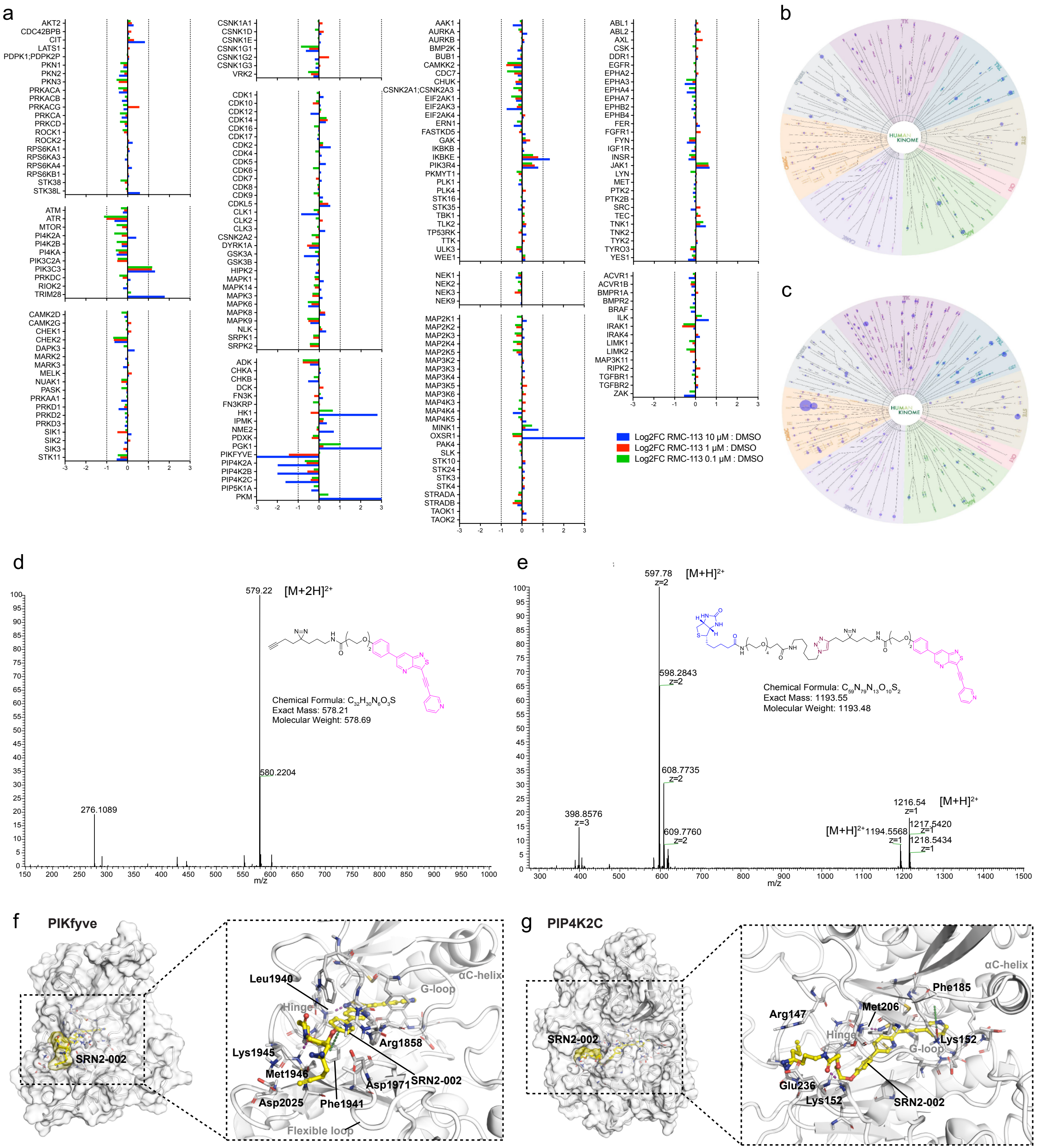

Supplementary Fig. 3

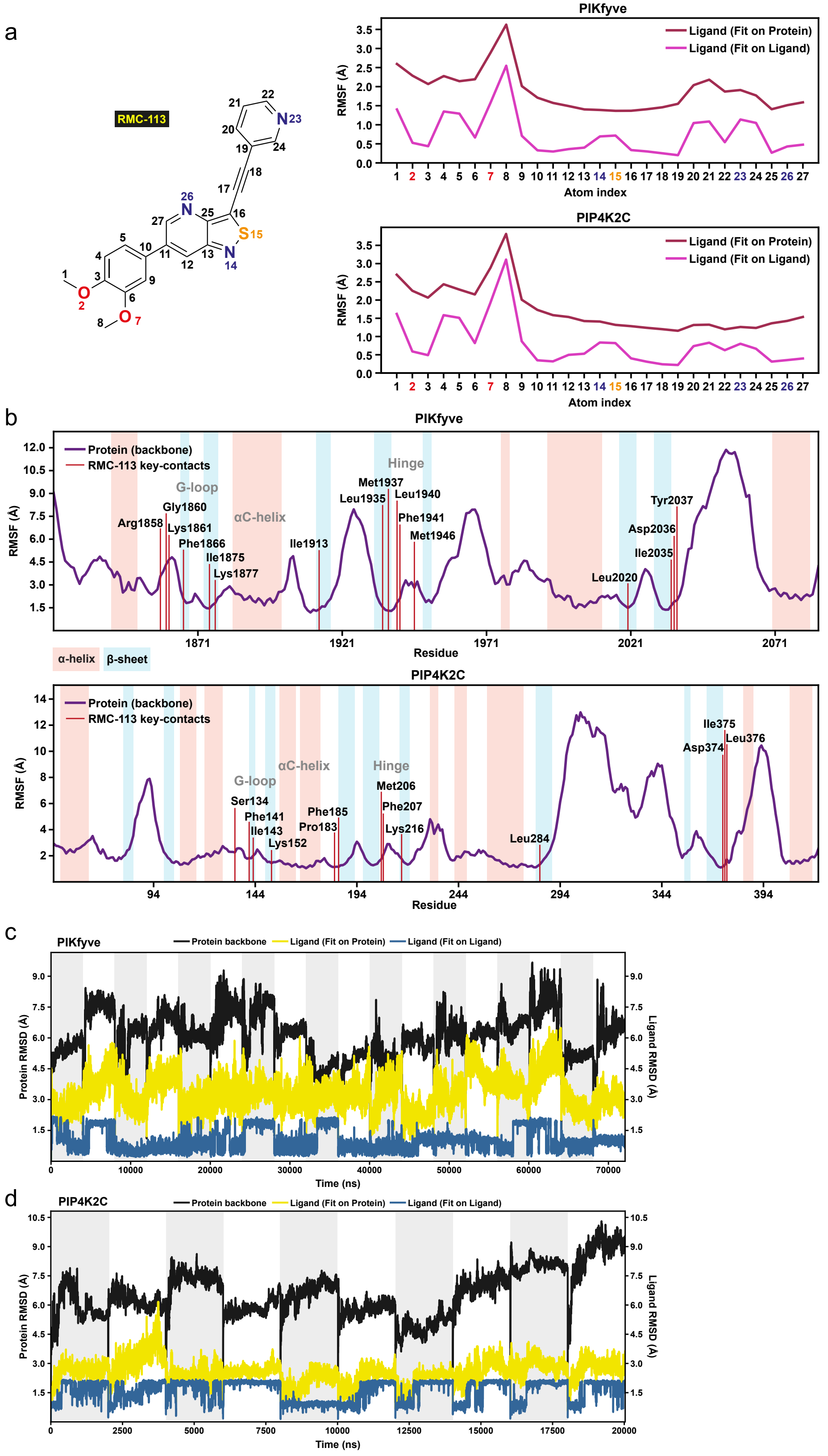

Supplementary Fig. 4

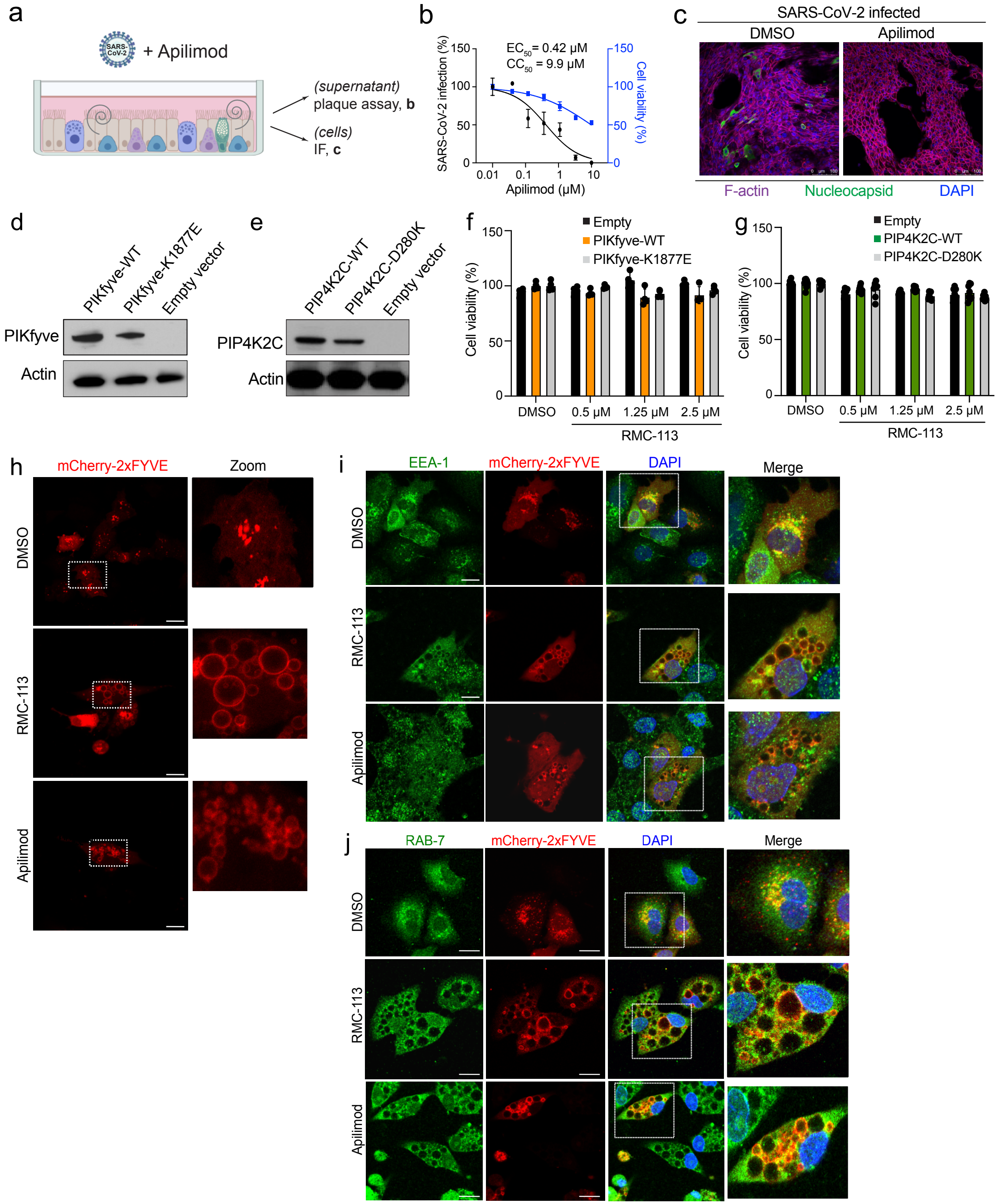

Supplementary Fig. 5

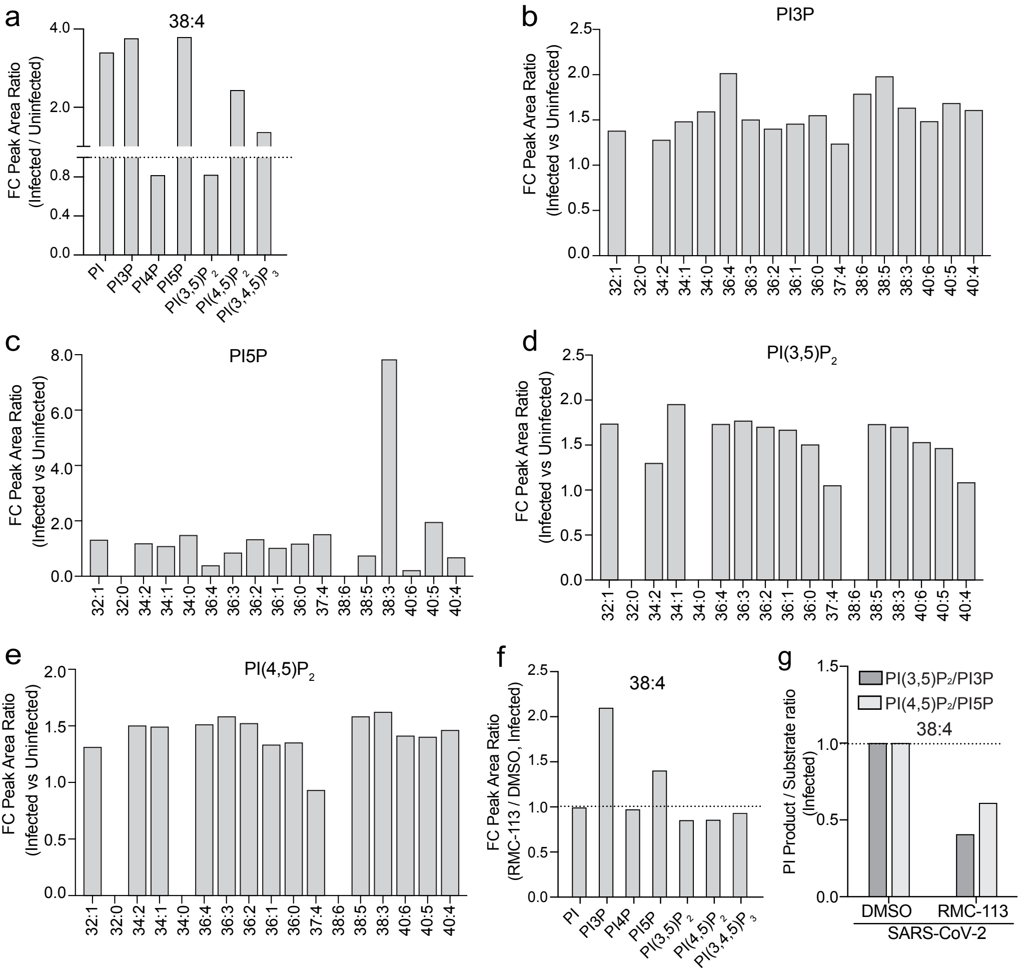

Supplementary Fig. 6

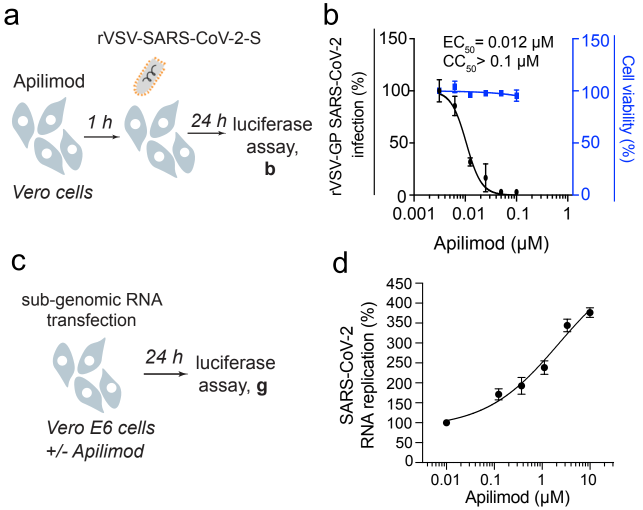

Supplementary Fig. 7

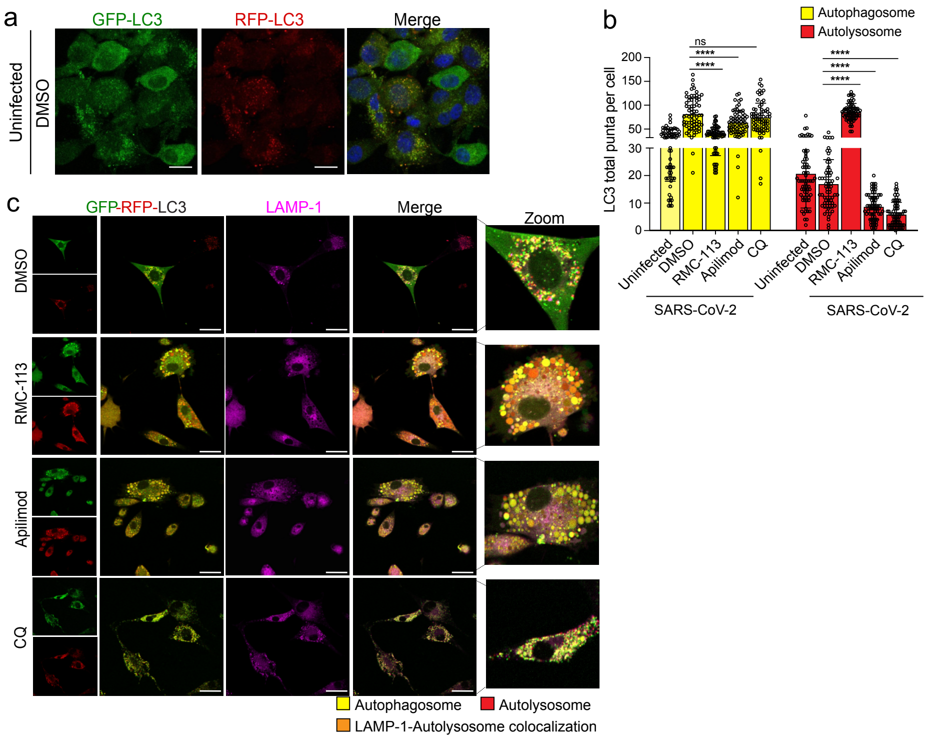

Supplementary Fig. 8

a

Infection status	Treatment	VHCs		Bystanders
		high >3	low (1-3)	zero
SARS-CoV-2	DMSO	421	2649	5571
	RMC-113	115	1363	6388
Uninfected	DMSO	0	0	1369
	RMC-113	0	0	1833

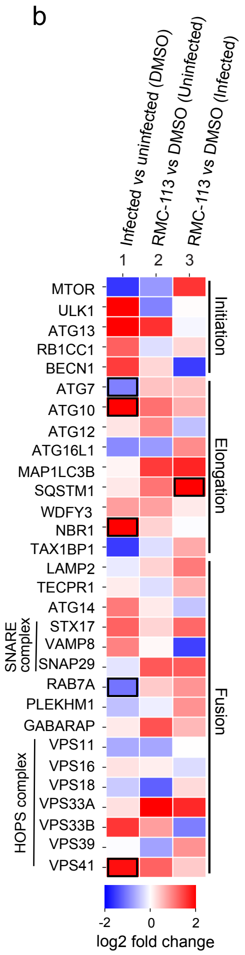

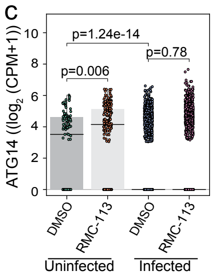

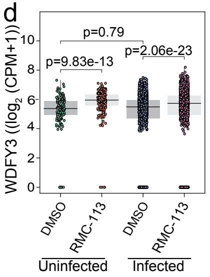

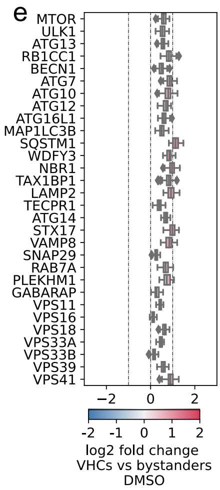

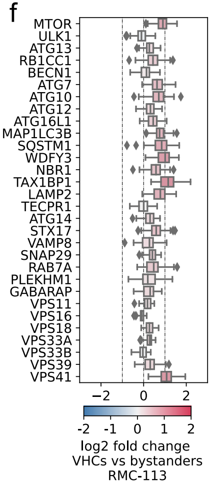

Supplementary Fig. 9

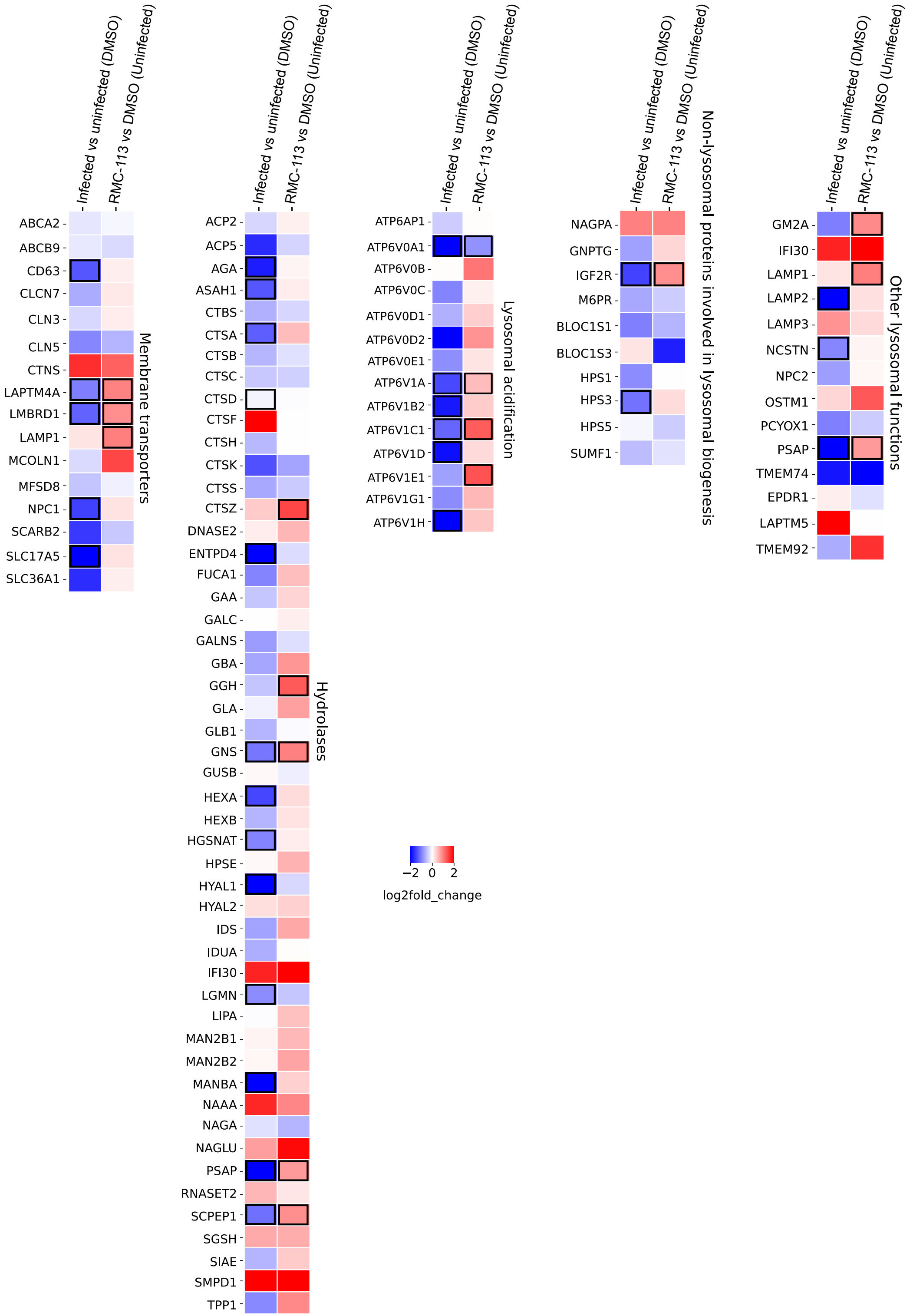

Supplementary Fig. 10

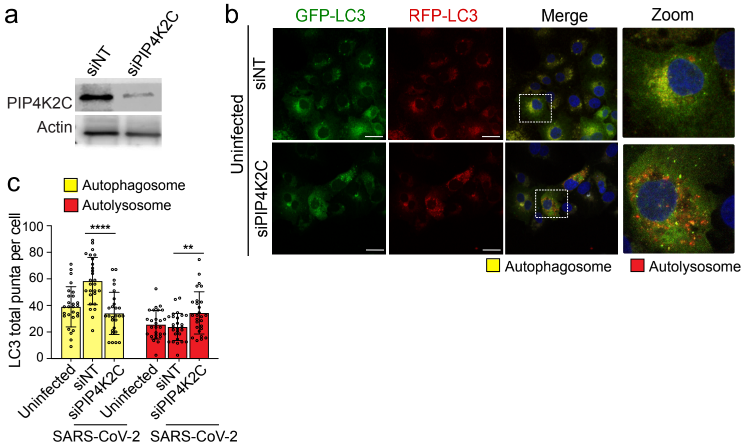

Supplement: Supplement 1 — Supplemental Fig. 1: RMC-113 has a broad-spectrum antiviral potential. a, Dose response to RMC-113 of rSARS-CoV-2-nLuc infection (black, USA-WA1/2020 strain, MOI = 0.05) and cell viability (blue) in Calu-3 cells via luciferase and alamarBlue assays at 24 hpi, respectively. b, Dose response to RMC-113 of rVSV-SARS-CoV-2-S infection (black) and cell viability (blue) in Calu-3 cells via luciferase and alamarBlue assays at 24hpi, respectively. c and d, Dose response of VEEV (TC-83) (MOI = 0.01) (c) and DENV2 (MOI = 0.1) (d) infections (black) and cell viability (blue) to RMC-113 measured in U-87 MG (c) and Huh7 (d) cells via luciferase and alamarBlue assays at 24 (c) and 48 (d) hpi, respectively. e and f, Dose response of EBOV (Kikwit isolate, MOI = 1) (e) and MARV (Ci67 strain, MOI = 2) (f) infections (black) and cell viability (blue) to RMC-113 measured in Huh7 cells via microneutralization and Cell-Titer-Glo luminescent assay at 48 hpi, respectively. Data are a combination (a-f) or representative (e, f) of two independent experiments with 2–5 replicates each. Means ± SD are shown. Data are relative to DMSO control. Supplemental Fig. 2: RMC-113 is a selective kinase inhibitor that stably binds PIKfyve and PIP4K2C. a, Label-free kinase abundance ratio between RMC-113 (0.1, 1.0, and 10 μM)- and DMSO-treated SUM159 cell lysates measured following affinity purification via multiplexed inhibitor beads kinome profiling and analyzed by mass spectrometry (MIB/MS). Shown are the Log2 fold change mean values ± SD of 2 replicates. b, Selectivity profiling of RMC-113 against 335 wildtype protein kinases at a concentration of 0.1 and 1 μM (ProQinase GmbH). Residual activity was determined relative to DMSO. The diameter of the dots reflects the % inhibition of the respective kinase. A selectivity score of 0.003 was measured. Data associated to ProQinase assays are shown in Table S1. c and d, Positive electrospray ionization (ESI) mass spectra showing: (c) the protonated molecular i [file media-1.pdf]
